# Investigation on the interlayer coupling and bonding in layered nitride-halides ThNF and ThNCl

**DOI:** 10.1039/d1ra05578j

**Published:** 2021-08-25

**Authors:** Xiao Liu, Da-Yong Liu, Ting-Ting Li, Dong-Meng Chen, Liang-Jian Zou

**Affiliations:** Key Laboratory of Materials Physics, Institute of Solid State Physics, HFIPS, Chinese Academy of Science Hefei 230031 China zou@theory.issp.ac.cn dyliu@theory.issp.ac.cn; Science Island Branch of the Graduate School, University of Science and Technology of China Hefei 230026 China; College of Science, China University of Petroleum Qingdao 266580 China

## Abstract

Motivated by recent experimental observation [N. Z. Wang, *et al.*, Inorg. Chem., 2019, **58**, 9897], we investigated the electronic properties and chemical bonding in layered nitride-halide compounds ThNF and ThNCl using first-principles calculations to illustrate the interlayer interaction. The energy gaps and chemical valences of both compounds are in agreement with experimental data. The crystal orbital Hamiltonian population (COHP) and charge density differential analysis show that interlayer chemical bonding plays a more important role than that van der Waals interactions in ThNF and ThNCl, in contrast to isostructural ZrNCl and HfNCl. These results explain why it is difficult to intercalate ThNF and ThNCl with charged particles, as observed in experiments.

## Abstract 2

Showing that the interlayer charge transfer and chemical bond in ThNF and ThNCl are considerable larger than the *van der Waals* compound ZrNCl, addressing the difficulty in intercalating Li ion or other charged molecules.

## Graphical abstract

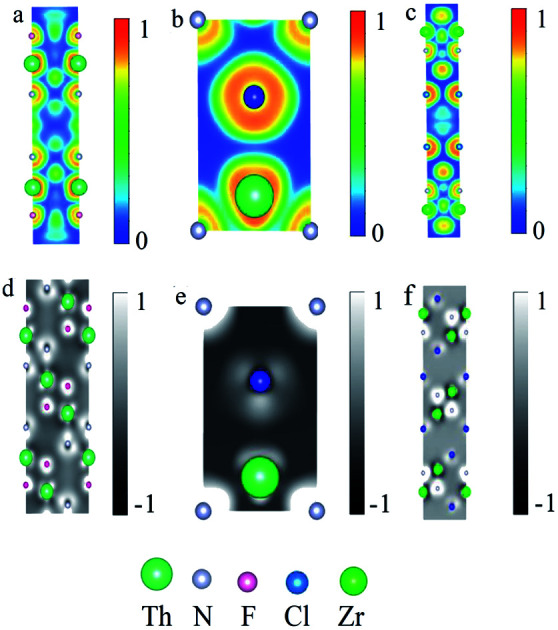

## I. Introduction

Two dimensional layered materials have attracted great interest since the discovery of 2D graphite–graphene in 2004.^1^ Since then, many 2D layered materials, such as new transition metal dichalcogenides (TMDS) and black phosphorus have been synthesized.^2,3^ These 2D layered materials offer a new platform to explore fundamental physics and chemistry in superconductivity, charge density waves, half-metallic magnetism and quantum transport.^4–6^ The interlayer interactions of these 2D materials are usually van der Waals coupling, and because of the weak interlayer coupling, one can easily modify the physical and chemical properties through pressure, intercalation, liquid and solid gating, and so on. Through physical property modification, and also that of their heterostructures, these 2D layered materials demonstrate great potential applications in novel devices.^7–10^

As 2D layered materials, the nitride fluorides and the nitride chlorides were studied early.^11^ ZrNCl, HfNCl and ZrNBr could be superconducting *via* lithium or organic molecule intercalation.^12–16^ Naturally, it comes to mind that whether layered nitride-halides ThNF and ThNCl can be superconducting or not through intercalation. Such attempts have been performed recently.^17^ Wang *et al.* found ThNF and ThNCl could not be intercalated by lithium or organic molecules like other nitride-halides ZrNCl and HfNCl. This arises a question that what happen of interlayer coupling and chemical bonding in these two compounds.

In order to illustrate why ThNF/Cl are so different from ZrNCl and HfNCl, we perform a first-principle electronic structure calculation study on the electronic structures and chemical bonding analysis, and show that the interlayer chemical bonding in ThNF/Cl is stronger than that in ZrNCl/HfNCl, and the van der Waals interaction is less important in ThNF/Cl. In the rest of this paper, we first optimize the crystal structures of ThNF and ThNCl to give the atomic positions, then present their electronic properties, the charge transfer, chemical valence and bonding in ThNF/Cl, and finally we summarize our results.

## II. Theoretical methods and numerical results

### (A) Structure optimizations

**Fig. 1:**
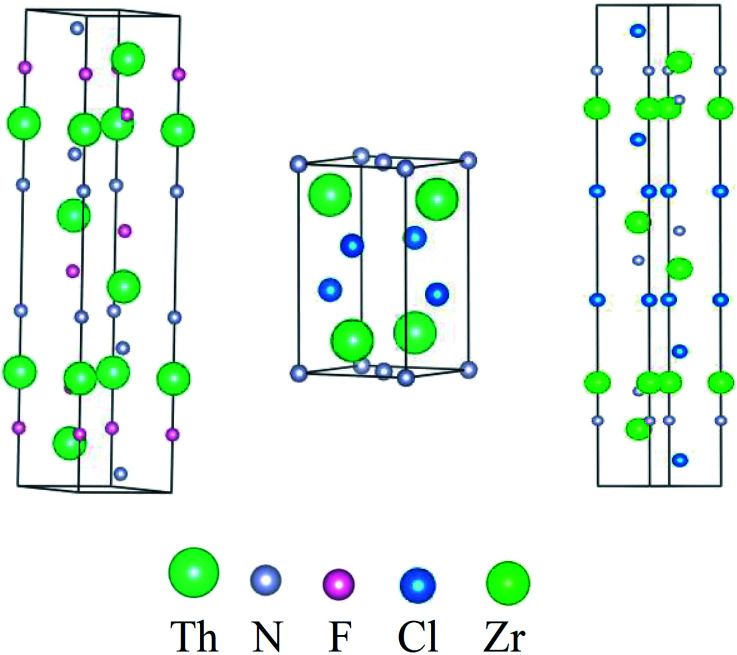
Crystal structures of ThNF, ThNCl and ZrNCl, respectively, for comparison.

In the 1960s, Juza^18^*et al.* synthesized a series of thorium nitride-halides ThNX (X = F, Cl, Br, I) and found that ThNF shows a rhombohedral symmetry and belongs to the *R*3̄*m* space group, while ThNCl crystallizes in tetragonal symmetry and belongs to the *P*4/*nmm* space group; meanwhile, the space group of ZrNCl is the same as ThNF. Fig. 1 displays their crystal structures and Table 1 summarizes the information of crystal structures of ThNF and ThNCl, as well as ZrNCl for comparison. Though the crystal parameters of ThNF/Cl are known, the atomic positions of these compounds are not well defined due to the lattice distortion.^19^

**Table 1:** Summary of crystallographic structure data of ThNF, ThNCl and ZrNCl

	ThNF	ThNCl	ZrNCl
*a* (Å)	4.009	4.097	3.568
*b* (Å)	4.009	4.097	3.568
*c* (Å)	20.239	6.895	27.672
*α*	90	90	90
*β*	90	90	90
*γ*	120	90	120
Volume (Å^3^)	281.7	115.74	302.33
Space group	*R*3̄*m*	*P*4/*nmm*	*R*3̄*m*

Through this paper, all DFT calculations were performed using the Vienna *ab initio* simulation package (VASP),^20–22^ the interaction between the ionic core and the valence electrons were described using the projection-augmented-wave (PAW) method using a cutoff energy of 520 eV. As for the exchange-correlation functional, the Perdew–Burke–Ernzerhof (PBE) generalized-gradient approximation (GGA) method were used in our calculation.^23^ Since the interactions between ThNF/ThNCl layers are of van der Waals (vdW) type, a more accurate method of non-local vdW-DF functional was adopted in our calculation, and the more accurate exchange functional optPBE-vdW was used.^24–27^

We first optimize the crystal structures of ThNF, ThNCl and ZrNCl, with considering the presence and absence of interlayer van der Waals interaction. We find that the planar lattice parameters *a* and *b* change little, the most significant effect is associated with the variation of lattice parameter *c* of these compounds, as seen in Table 2.

**Table 2:** Comparison of the experimental *c*-axis lattice parameter with optimized results without and with van der Waals corrections

	Exp. data	Without vdW	With vdW	Difference o/w vdW
ThNCl	6.895	7.040	6.952	2.1%/0.8%
ThNF	20.229	20.498	20.368	1.3%/0.7%
ZrNCl	27.672	29.571	27.699	6.81%/0.1%

Comparing the optimization results with experimental data, we find that without consideration the vdW correction, the difference of the optimized *c*-parameter from experimental data for ThNCl, ThNF and ZrNCl are 2.1%, 1.3%, and 6.81%, respectively. Once with the vdW correction, the difference reduces to 0.8%, 0.7%, and 0.1%, respectively, as shown in the last column of Table 2. This demonstrates that the interlayer vdW interaction in ThNF/ThNCl is much weaker than in ZrNCl. This arises an interesting question why the interlayer van der Waals interaction plays a less role in ThNF/ThNCl.

### (B) Electronic properties

**Fig. 2:**
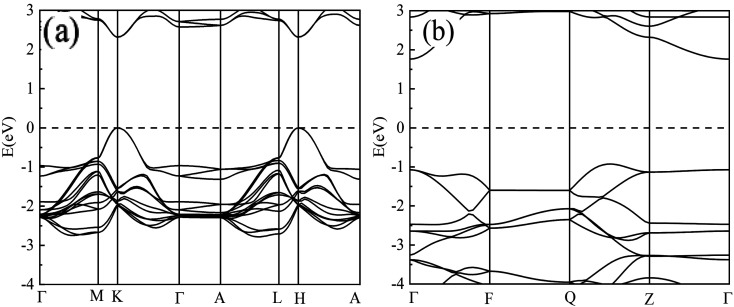
Band structures of ThNF (a) and ThNCl (b), respectively.

To uncover why the van der Waals potential does not play a crucial role in ThNCl and ThNF, we perform the electronic structure calculation on them, as well as ZrNCl for comparison. The band structures of ThNCl, ThNF, and ZrNCl were calculated by the *ab initio* quantum mechanical software package VASP code, the 13 × 13 × 3 and 7 × 7 × 5 Monkhorst–Pack meshes were used for *k*-point sampling with the Brillouin zones of the ThNF and ThNCl unit cells, respectively. The band structures obtained are shown in Fig. 2. From Fig. 2, ThNF and ThNCl are direct and indirect semiconductors with band gaps of 2.3 eV at K point, and 2.8 eV from *Γ* point to valence band maximum between *Q* and *Z*, respectively. As a comparison, the experimental data showed that the band gaps are ∼3.0 eV for ThNF^17^ and 3.79 eV for ThNCl,^17^ respectively. The theoretical band gaps are comparable with, but considerably smaller than experimental data.

### (C) Charge transfer and chemical valence

To further analyze the interlayer couplings in ThNCl and ThNF, we have also calculated the charge transfer in ThNCl and ThNF, as well as ZrNCl, by using the atoms in molecules (AIM) approach, which splits molecules into atoms as based purely on the electronic charge density after the self-consistent calculations through VASP. This analysis is useful in evaluating charge transfer and chemical valence. Here we find that in ThNF, the valence of each element is *Q*(Th) = +2.3, *Q*(N) = −1.43, *Q*(F) = −0.87; and in ThNCl, *Q*(Th) = +2.38, *Q*(N) = −1.62, *Q*(Cl) = −0.76, in agreement with the experimental data by the X-ray photoemission spectra (XPS) very well.^17^

**Fig. 3:**
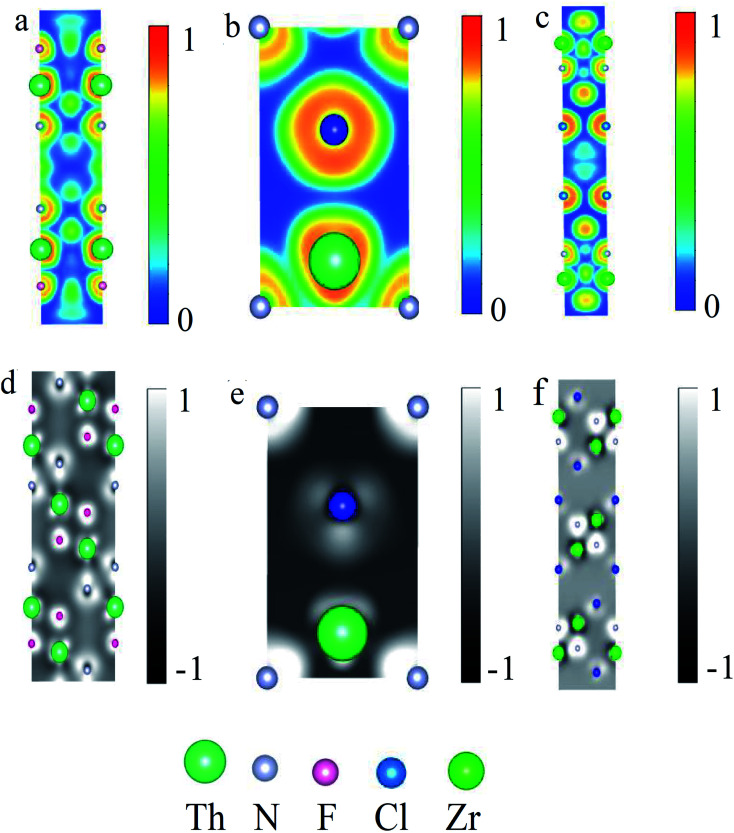
Electron localization functions (a–c) and charge deformation density (d–f) of ThNF, ThNCl and ZrNCl, respectively. Slices in electron localization function are (100) face, slices in charge deformation density are (110) face for ThNF and ZrNCl and (100) face for ThNCl, respectively. In (d–f) the 1 and −1 denote the relative magnitudes of the gain and loss charges.

The charge analysis of ThNCl and ThNF, as well as ZrNCl, gives rise to their electron localization function and deformation charge density, as shown in Fig. 3. Fig. 3a shows that in the ThN intralayer, the electron localization function of ThNF displays an obvious overlap of charge density between Th ions and N ions; as a comparison, one observes almost negligible overlap between Th and F, as seen in Fig. 3a, showing the formation of chemical bonding between Th and N. Similar results are also seen in ThNCl and ZrNCl in Fig. 3b and c, the Th–N/Zr–N strongly bonds but Th–Cl does not bond, while Zr–Cl weakly bonds. This is consistent with the charge transfer result in the last paragraph. On the other hand, the deformation charge density analysis shows that Th and N are valence bonded in ThNF and ThNCl, but Th–F/Th–Cl are ionic bonded in ThNF and ThNCl, as seen in Fig. 3d and e. As a comparison, both Zr–Cl and Zr–N are ionic bonded in ZrNCl, as seen in Fig. 3f. These results lead to distinct difference of the interlayer interactions among ThNCl, ThNF and ZrNCl.

Moreover, in the deformation charge density from Fig. 3d–f, we can see that the interlayer charge transfers in ThNF and ThNCl are considerably different from that in ZrNCl. From Fig. 3d, we can see that considerable charge of Th atoms (the green atoms) transfers to F atoms between the intralayer Th–F and interlayer Th–F, leading to the bonding between different ThNF layers is tightly. Similar situation also occurs in Th–Cl of ThNCl shown in Fig. 3e when multiple layers are considered. As a comparison of ZrNCl in Fig. 3f, one finds that the charge transfer between Zr and Cl in ZrNCl slabs is quite different: the charge transfer mainly occurs in intralayer Th–N in ZrNCl slabs, the interlayer charge transfer is small, suggesting that the interlayer interaction in ZrNCl is typical van der Waals coupling. This is why ZrNCl can be easily intercalated but ThNF/ThNCl can not. To further confirm our argument, we calculate the interlayer interaction energy and the exfoliated energy quantitatively in next subsection.

### (D) Chemical bonding

**Fig. 4:**
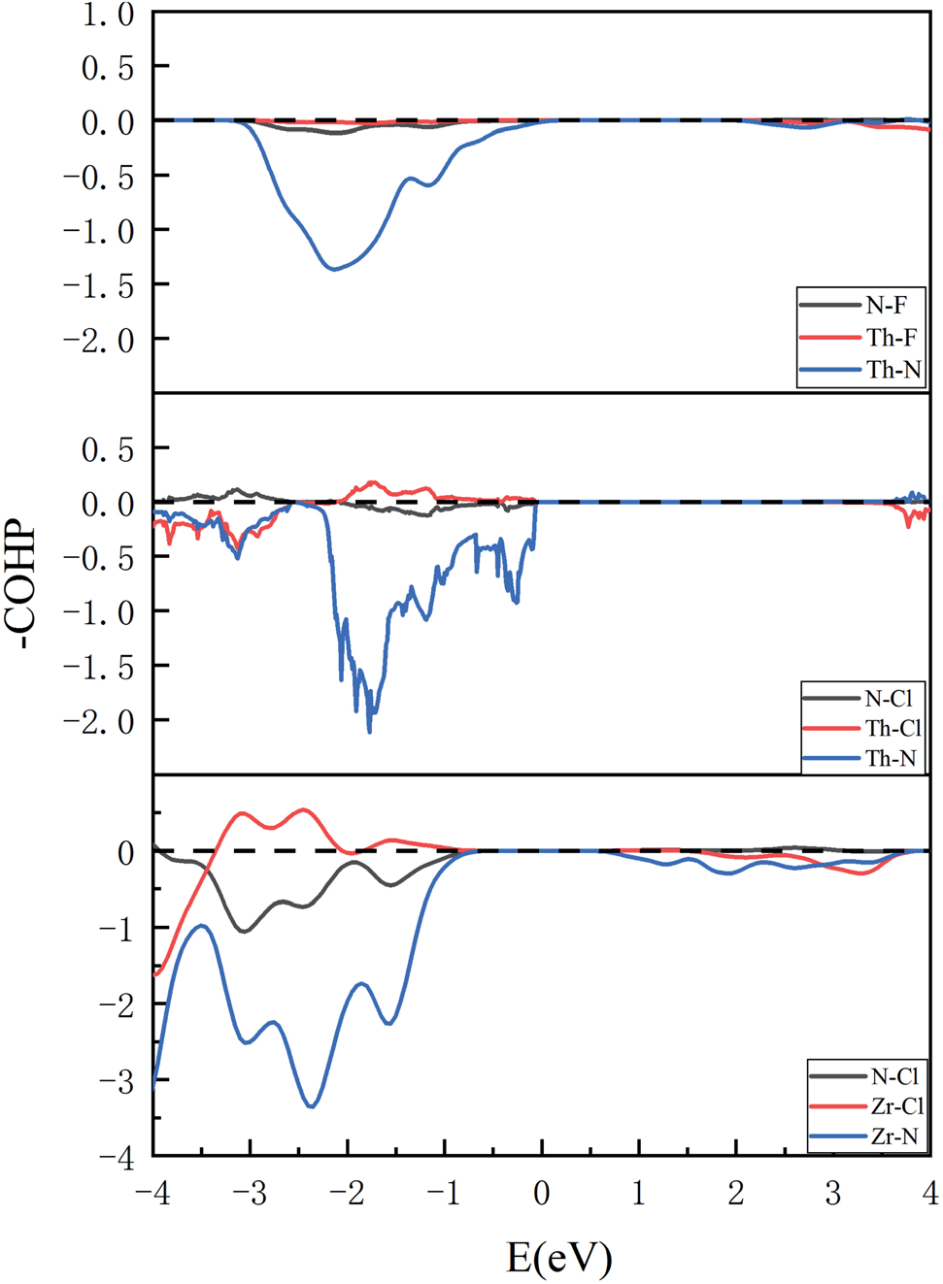
COHP results show bonding situations between different elements in ThNF, ThNCl, and ZrNCl, respectively.

The quantum hybridization among elements Th–N, Th–F and Th–Cl in ThNF/ThNCl can be further assessed using the crystal orbital Hamilton population (COHP), which is a theoretical bond-detecting tool for solids. In what follows we furthermore address the relative strengths of the bonds in ThNCl/ThNF using the integrated COHP (iCOHP). Fig. 4 plots the COHP of intralayer atoms in ThNF and ThNCl. From Fig. 4, one sees that Th–N contributes most dominant COHP, suggesting a common fact that the metallic ions are strongly bonded with N; comparatively, N–F/N–Cl and Th–F/Th–Cl are weakly bonded.

In order to uncover the interlayer bonding in ThNF and ThNCl, we first calculate the interlayer interaction energy (IE) and the exfoliation energy which are important to describe the chemical properties of layer materials.^28,29^ We find that the IEs are −82.29, −71.28, and −17.48 meV per atom for ThNF, ThNCl and ZrNCl, respectively. Comparing with the IE values of black phosphorus and black As are −38.76 and −54.61, meV per atom, respectively reported by F. Sato, *et al.*,^28^ we can see that the bondings of ThNF slabs and ThNCl slabs are stronger than ZrNCl, black phosphorus and black As. And we also calculated the exfoliation energy which is important to describe the interlayer interaction in layer compounds. We find that the exfoliation energies are 0.0719, 0.057, and 0.016 eV Å^−2^ for ThNF, ThNCl, and ZrNCl, respectively, implying that ThNF and ThNCl are more difficult to be exfoliated than ZrNCl, in consistent with the IE results. These results further confirm interlayer couplings of ThNF/ThNCl are stronger than ZrNCl, thus not easily intercalate charged particles.

**Fig. 5:**
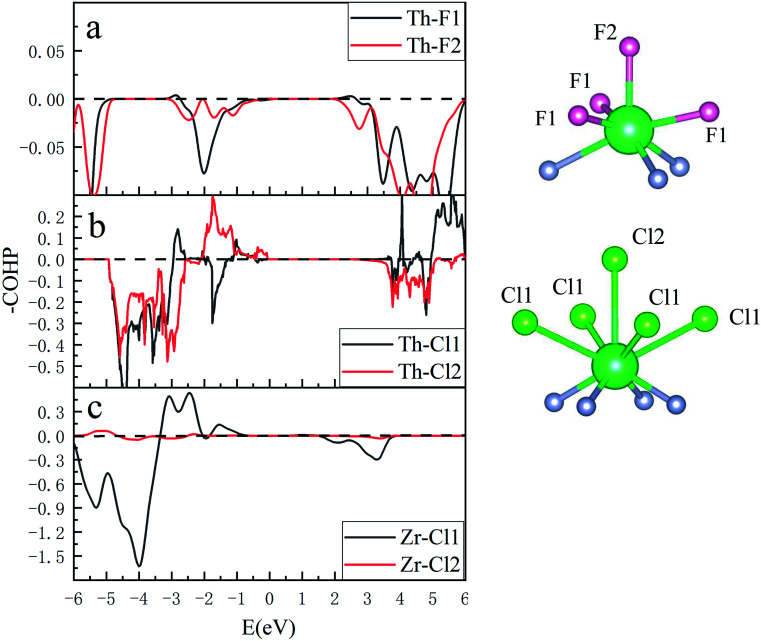
(left) Chemical bonding of Th–F (a), Th–Cl (b) and Zr–Cl (c) in intralayer and interlayer shown in COHP diagram. (right) Sketches of intralayer and interlayer Th–F labeled as F_1_ and F_2_ and Th–Cl labeled as Cl_1_ and Cl_2_, respectively.

Moreover, we calculate and compare the interlayer COHP of ThNCl and ThNCl, as well as ZrNCl for comparison, in Fig. 5. We first notice that the distances of Th–F_1_ and Th–F_2_ in ThNF, Th–Cl_1_ and Th–Cl_2_ in ThNCl, are 2.622 Å, 2.624 Å, 3.156 Å and 3.259 Å, respectively; here 1 and 2 denote the intralayer and interlayer sites, as shown in Fig. 5; by contrast, the distances of Zr–Cl_1_ and Zr–Cl_2_ in ZrNCl are 2.772 Å and 5.214 Å, respectively, showing that the void distance of ZrNCl is greatly larger than those of ThNF and ThNCl.

Correspondingly, one sees from Fig. 5a that −COHP of both Th–F_1_ and Th–F_2_ are comparable, showing that both Th–F_1_ and Th–F_2_ are in bonding states. For Th–Cl_1_ and Th–Cl_2_ in ThNCl, the major contributions below *E*_F_ in Fig. 5c suggest that both Th–Cl_1_ and Th–Cl_2_ are also in bonding states. As a comparison, Zr ions are bonded strongly with the intralayer Cl ions and are not bonded to the interlayer Cl ions, as seen in Fig. 5b. These further prove that the interlayer chemical bonding between ThNF and ThNCl is stronger than that in ZrNCl. Therefore, the van der Waals coupling in ZrNCl plays more important roles than in ThNCl and ThNF, leading to easy intercalation behavior in the ZrNCl and hard intercalation behavior in the ThNF and ThNCl, as observed in the experiment.^17^

## III. Conclusion

In summary, with considering the van der Waals correction, the optimization result is confirming almost exactly to the experimental data for ZrNCl, however, theoretical results and experiment cannot fit well for ThNF and ThNCl. In order to illustrate this phenomenon, we perform further investigations show their charge transfer, electronic properties and chemical bonding. Unlike ZrNCl, van der Waals interaction plays a less important role in ThNF and ThNCl, the interlayer chemical bonds are strong leading that intercalation is more difficult in ThNF and ThNCl than in ZrNCl, gives a good interpretation the different intercalation behavior of ThNF, ThNCl and ZrNCl, respectively.

## Conflicts of interest

There are no conflicts to declare.

## Supplementary Material
